# Nile Tilapia Derived Antimicrobial Peptide TP4 Exerts Antineoplastic Activity Through Microtubule Disruption

**DOI:** 10.3390/md16120462

**Published:** 2018-11-22

**Authors:** Chen-Hung Ting, Yi-Chung Liu, Ping-Chiang Lyu, Jyh-Yih Chen

**Affiliations:** 1 Marine Research Station, Institute of Cellular and Organismic Biology, Academia Sinica, Ilan 262, Taiwan; koichiting@gmail.com; 2 Institute of Population Sciences, National Health Research Institutes, Zhunan 350, Taiwan; jong212@gmail.com; 3 Institute of Bioinformatics and Structural Biology, National Tsing-Hua University, Hsinchu 300, Taiwan; pclyu@mx.nthu.edu.tw; 4 The iEGG and Animal Biotechnology Center, National Chung Hsing University, Taichung 402, Taiwan

**Keywords:** antimicrobial peptide (AMP), tilapia piscidin 4 (TP4), microtubule

## Abstract

Some antimicrobial peptides (AMPs) exhibit anti-cancer activity, acting on cancer cells either by causing membrane lysis or via intracellular effects. While intracellular penetration of AMPs has been shown to cause cancer cell death, the mechanisms of toxicity remain largely unknown. Here we show that a tilapia-derived AMP, Tilapia piscidin (TP) 4, penetrates intracellularly and targets the microtubule network. A pull-down assay identified α-Tubulin as a major interaction partner for TP4, and molecular docking analysis suggested that Phe1, Ile16, and Arg23 on TP4 are required for the interaction. TP4 treatment in A549 cells was found to disrupt the microtubule network in cells, and mutation of the essential TP4 residues prevented microtubule depolymerization in vitro. Importantly, the TP4 mutants also showed decreased cytotoxicity in A549 cells, suggesting that microtubule disruption is a major mechanistic component of TP4-mediated death in lung carcinoma cells.

## 1. Introduction

Antimicrobial peptides (AMPs) are found in many species, including a wide variety of plants and animals [1]. The endogenous functions of these molecules are associated with both innate and adaptive immunity, and their expression may be induced in response to pathogen infection or to modulate the immune response [2]. Based on these functions, synthesized short AMPs have been suggested as potential anti-infective agents that may provide alternatives to antibiotics [3,4,5,6,7]. Many AMPs disrupt bacterial membranes via pore formation or a detergent-like action [8], while others operate via membrane non-lytic mechanisms [9]. Interestingly, a few synthesized AMPs have been shown to be cytotoxic to both antibiotic-resistant bacteria [4,5,6] and cancerous cell lines [10,11,12,13,14,15,16,17,18]. Cancer cells often harbor negatively charged plasma membranes, which attract cationic AMPs through electrostatic interactions [11,17,18]. The AMPs then kill cancer cells directly via membrane-lytic pathways and/or after intracellular penetration [11,13,15]. Once inside the cells, AMPs have been shown to partly accumulate in certain organelles and induce apoptotic or necrotic cell death [11,13,19]; however, the molecular targets of these cytotoxic molecules have not been previously identified.

TP4 was identified in Nile tilapia (*Oreochromis niloticus*) [20]. In that study, five piscidin genes, named *TP1-5*, were found in a screen of cDNA clones from the Nile tilapia spleen [20]. Among the gene products, a short TP4-based peptide showed especially robust activity as a broad spectrum bacterial-killing agent [20]. Synthesized TP4 was shown to efficiently kill methicillin-resistant *Staphylococcus aureus* (MRSA), while also stimulating cell proliferation and tissue recovery in infected wounds in mice [4]. In addition to its function as an antimicrobial agent, TP4 also exerts excellent anti-tumor activity both in vitro and in vivo [11]. The mechanisms underlying this anticancer activity have been reported to involve cellular uptake and the induction of mitochondrial and calcium homeostatic stresses, which trigger necrotic death [11]. Another report indicated that TP4 may induce apoptosis via activation of extrinsic Fas/FasL and intracellular intrinsic mitochondria-mediated pathways in osteosarcoma MG63 cells [10]. However, the intracellular targets of TP4 and the mechanisms leading to cell death remain largely uncharacterized. In the present work, we aimed to identify intracellular targets of TP4 and evaluate the cellular effects of TP4-target interactions in A549 lung cancer cells, which are known to experience cytotoxicity after intracellular uptake of TP4. An in vitro pull-down assay, combined with LC-MS/MS, identified α-Tubulin as an intracellular target of TP4. Molecular docking, mutation studies, immunocytochemistry, and cell viability assays were then performed to validate the interaction and characterize TP4-induced microtubule disruption.

## 2. Results

### 2.1. Synthesized TP4 binds to Tubulin

**Figure 1:**
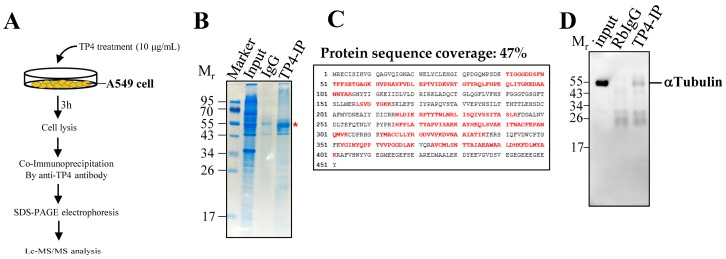
TP4 interacts with α-Tubulin. (**A**) Procedure showing the pull down of TP4 interaction partners with TP4 antibody. (**B**) Coomassie blue-stained SDS-PAGE shows a major band at about 55 kDa is pulled down by the TP4 antibody. Input lane has total lysate before co-immunoprecipitation (IP). IgG lane shows IP using rabbit IgG. TP4-IP lane shows IP using TP4 antibody. * Red star indicates the protein band that was excised for in-gel digestion followed by LC-MS/MS analysis. (**C**) Protein database searching of peptides detected by MS identified α-Tubulin as a TP4 binding partner. α-Tubulin sequence is shown. Red letters indicate peptides identified in the MS analysis. (**D**) Immunoblotting analysis using antibody against α-Tubulin. Mr is the molecular weight.

To characterize the intracellular targets of TP4, cell lysates from A549 cells treated with 10 μg mL^−1^ TP4 for 3 h were pulled down with the TP4 antibody, see Figure 1A. SDS-PAGE analysis of the pull-down samples revealed a major band at approximately 55 kDa, as shown in Figure 1B. The dominant band was excised from the gel and analyzed by LC-MS/MS. Based on a MASCOT search for peptides found by MS, the potential binding target for TP4 was identified as α-Tubulin, see Figure 1C and Supplementary Dataset S1. Immunoprecipitation (IP) protein samples were then probed with α-Tubulin antibody, confirming the interaction between TP4 and α-Tubulin in A549 cells in vivo, see Figure 1D. Because tubulin is the major component of microtubules, we hypothesized that the cytotoxic effects of intracellular TP4 may be related to microtubule dynamics.

### 2.2. TP4 Disrupts the Microtubule Network in A549 Cells

**Figure 2:**
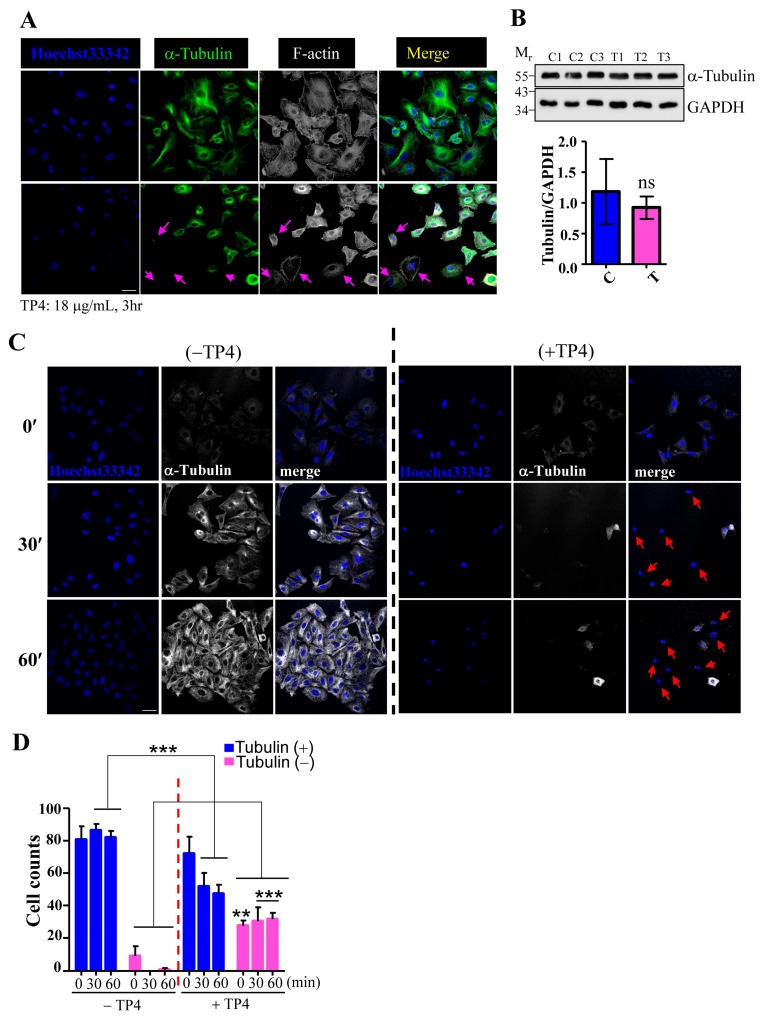
TP4 disrupts the microtubule network in cancer cells. (**A**) Cells were stained for α-Tubulin and F-actin. Hoechst33342 was used to stain nuclei. Arrows indicate cells with negative α-Tubulin staining. Bar: 20 μm. (**B**) Total lysates from cells with mock (labeled C) or TP4 treatment (labeled T) were analyzed by Western blot using antibodies against GAPDH and α-Tubulin. Quantification of the α-Tubulin signal normalized to GAPDH is shown. Mr is the molecular weight. (**C**) A549 cells pretreated with 20 μM nocodazole were stained for α-Tubulin (white) at 0, 30, and 60 minutes after nocodazole washout. Hoechst33342 was used to stain nuclei (blue). Arrows indicate cells with defective microtubule regrowth. Bar: 20 μm (**D**) Quantification of the number of cells with Tubulin-positive and Tubulin-negative staining. Results are presented as mean ± SD. *N* = 3, two-way analysis of variance (ANOVA): **p < 0.05; ***p < 0.001.

To examine whether TP4 may affect microtubule dynamics, A549 cells were treated with TP4, and cytoskeletal elements were analyzed by immunocytochemistry (ICC). The results showed that the microtubule network, but not F-actin, is clearly affected by TP4 treatment in A549 cells, see Figure 2A, indicated by arrows. We then asked whether TP4 treatment may influence tubulin protein levels by performing Western blots on lysates from A549 cells that had been treated with vehicle or TP4. The level of α-Tubulin protein was not significantly affected by TP4 treatment, as shown in Figure 2B, suggesting that intracellular TP4 may function by directly disrupting the microtubule network and not by modulating tubulin expression or stability. We next conducted a microtubule regrowth assay to further address whether TP4 may disrupt microtubule polymerization. TP4 was applied to A549 cells that had been treated with or without Nocodazole (20 μM) treatment for 6 h, which completely depolymerizes the microtubule network. The repolymerization of microtubules was then initialized by the addition of fresh recovery medium, and regrowth was analyzed by ICC. Almost all cells without TP4 treatment showed obvious microtubule regrowth after the addition of recovery medium, while TP4-treated cells did not, see Figure 2C. After quantification, we found that an increased portion of TP4-treated cells had microtubule regrowth defects, see Figure 2D. These results suggest that intracellular TP4 causes defects in the microtubule cytoskeleton in cancer cells.

### 2.3. Molecular Docking Analysis of the TP4-Tubulin Interaction

**Figure 3:**
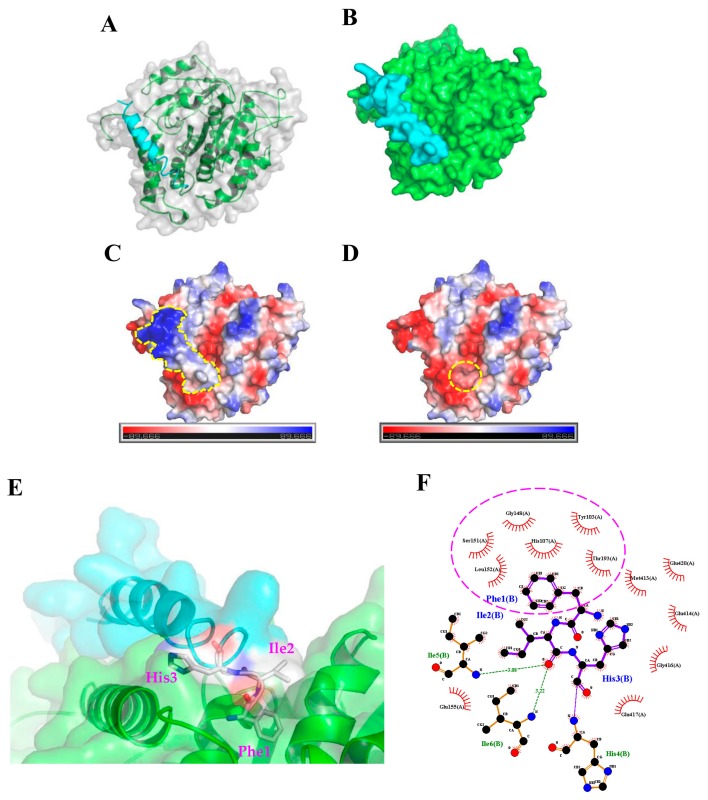
The first model from the molecular docking analysis of TP4-α-Tubulin interaction. (**A**,**B**) A three-dimensional structure is shown of predicted TP4 (cyan) binding to α-Tubulin (green). (**C**,**D**) Molecular surface electrostatic potentials of TP4/α-Tubulin complex are displayed. Blue and red colors represent regions of positive and negative charges, respectively. TP4 is encircled by a dashed line in (**C**). A dashed circle in (**D**) indicates the cavity in α-Tubulin that is predicted to interact with the Phe1 residue of TP4. (**E**) Magnified image showing how TP4 may dock in the α-Tubulin cavity. (**F**) The Phe1 residue in the TP4 is predicted to interact with Tyr103, His107, Gly148, Ser151, Leu152, and Thr193 of α-Tubulin through hydrophobic interactions (encircled by a dashed line).

**Figure 4:**
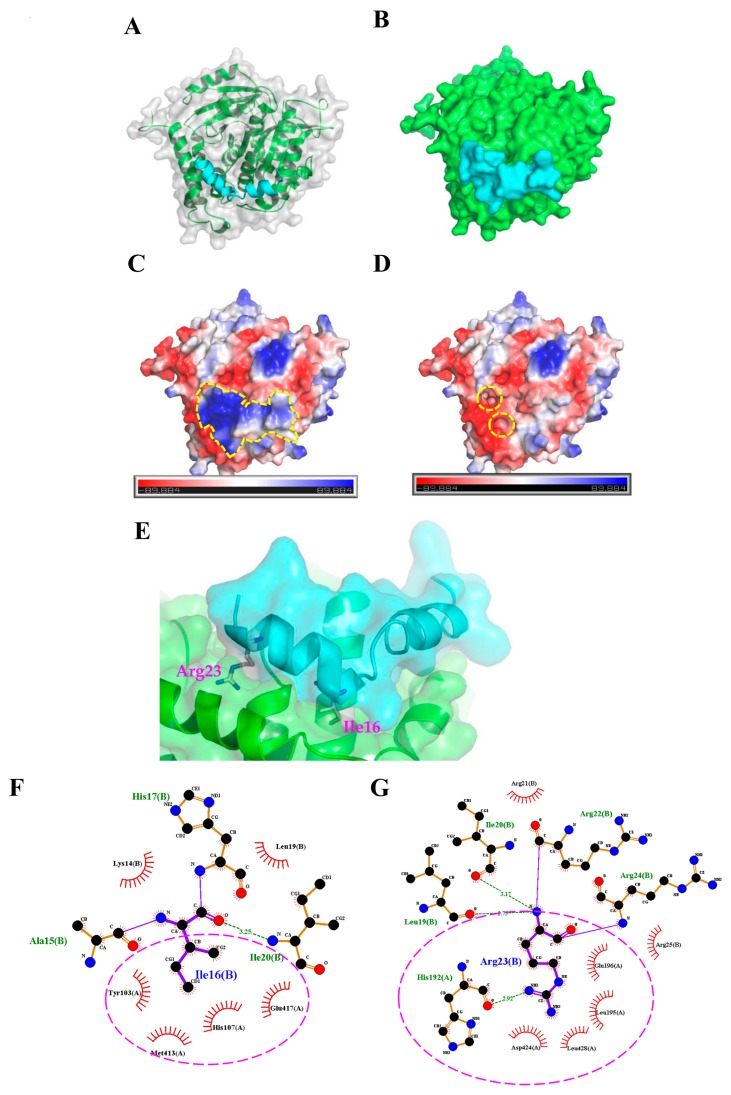
The second model from the molecular docking analysis of TP4-α-Tubulin interaction. (**A**,**B**) A three-dimensional structure is shown of the predicted TP4 (cyan) and α-Tubulin (green) interaction. (**C**,**D**) Molecular surface electrostatic potentials of TP4/α-Tubulin complex are displayed. Blue and red colors represent regions with positive and negative charges, respectively. TP4 is encircled by a dashed line in (**C**). (**D**) Dashed circles indicate the cavities in α-Tubulin that are predicted to participate in the TP4 interaction. (**E**) A magnified image shows how TP4 is predicted to dock in the cavities of α-Tubulin through the Ile16 and Arg23. (**F**,**G**) Ile16 of TP4 may interact with Tyr103, His107, Met413, and Glu417 residues of α-tubulin through hydrophobic interactions (**F**, encircled by a dashed line). Arg23 of TP4 may interact with Leu195, Glu196, Asp424, and Leu428 residues of α-Tubulin through hydrophobic interactions and with His192 residue through a hydrogen bond (**G**, labeled by a circle).

To further characterize how TP4 interacts with α-Tubulin, molecular docking analysis to evaluate potential interaction sites was conducted with the CABS-dock web server (http://biocomp.chem.uw.edu.pl/CABSdock). Two potential models were proposed by the program and are shown in Figure 3 and Figure 4. Model 1, see Figure 3A-C, predicts that the first phenylalanine (Phe1) of TP4 may insert into the cavity of α-Tubulin, see Figure 3D,E, where it is stabilized by Tubulin residues, Tyr103, His107, Gly148, Ser151, Leu152, and Thr193, through hydrophobic interactions, see Figure 3F. On the other hand, model 2, see Figure 4A-C, predicts that the Ile16 and Arg23 of TP4 may insert into two distinct α-Tubulin cavities, see Figure 4D,E. In this model, Ile16 interacts with the Tyr103, His107, Met413, and Glu417 through hydrophobic interactions, see Figure 4F. Meanwhile, Arg23 interacts with the Leu195, Glu196, Asp 424, and Leu428 through hydrophobic interactions and forms a hydrogen bond with His192, see Figure 4G; positively charged TP4 Arg23 and negatively charged α-Tubulin Glu196/Asp424 also contribute to electrostatic and steric stabilization.

### 2.4. TP4 Inhibits Microtubule Polymerization

**Figure 5:**
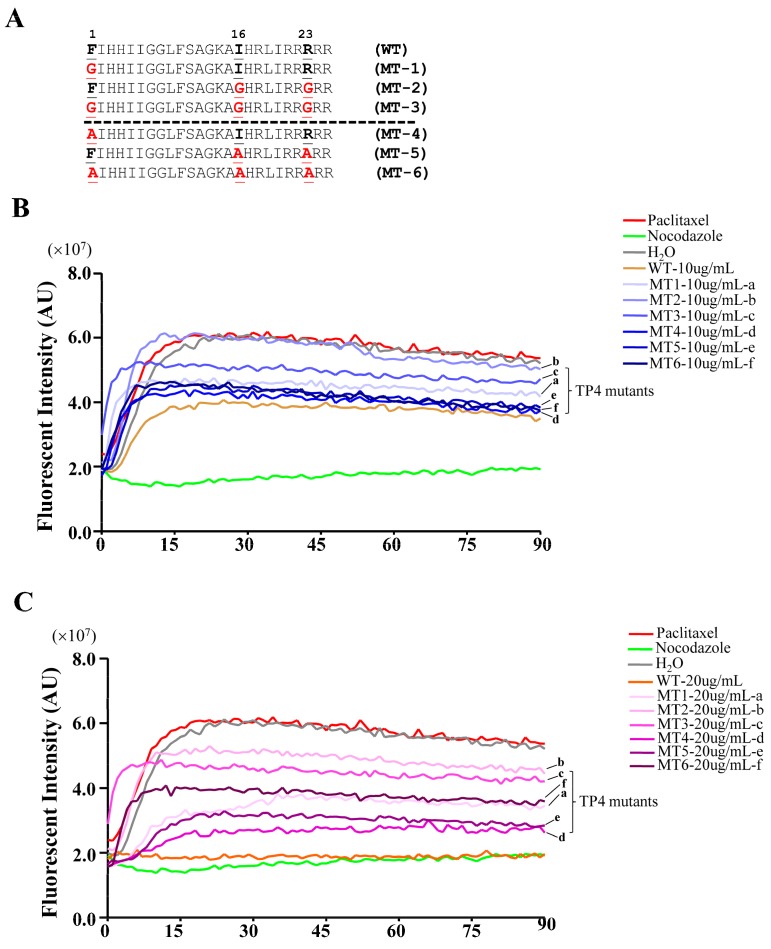
TP4 inhibits microtubule polymerization. (**A**) Sequences of the TP4 wild type (WT) and mutants. Letters shown in red indicate substituted amino acids. (**B**,**C**) Dynamic measurement of the microtubule polymerization. Pure tubulins were assembled in the presence of TP4 (WT), TP4 mutants (MT1-6). H_2_O, paclitaxel, and nocodazole served as vehicle, positive, and negative controls, respectively.

To test the models predicted by the molecular docking analysis, TP4 mutants were generated by substitution of Phe1, Ile16, and Arg23 with either Gly or Ala (mutant names and sequences are provided in Figure 5A), and the mutants were analyzed for their interaction with α-Tubulin. The sequence of TP4 is expected to form an α-helix, see Supplementary Figure S1A. Structural prediction of the TP4 mutants revealed that the amino acid substitution of Gly at positions 16 and 23 may disrupt the helical structure, while Ala substitution will not, see Supplementary Figure S1B–G. We next investigated whether TP4 mutants may disrupt tubulin polymerization by an in vitro tubulin polymerization assay. The result showed that 10 μg mL^−1^ of TP4 is sufficient to disrupt microtubule polymerization, see Figure 5B, and 20 μg mL^−1^ (a dose that causes obvious cell death) completely inhibited microtubule polymerization compared to controls, as shown in Figure 5C. All of the TP4 mutants showed a decreased ability to inhibit microtubule polymerization, but the effects were more obvious in Gly substitution mutants than Ala mutants, see Figure 5B,C, (a, b vs. d, f). In addition, mutations at Ile16 and Arg23 were more effective at mitigating the effects of TP4 on microtubule polymerization than Phe1 mutants, as shown in Figure 5B,C, (b, e vs. a, d). These results favor the interaction model 2, suggesting that Ile16 and Arg23 may play critical roles in the TP4-tubulin interaction. 

### 2.5. TP4 Mutants Exhibit Diminished Cancer Cell Killing

**Figure 6:**
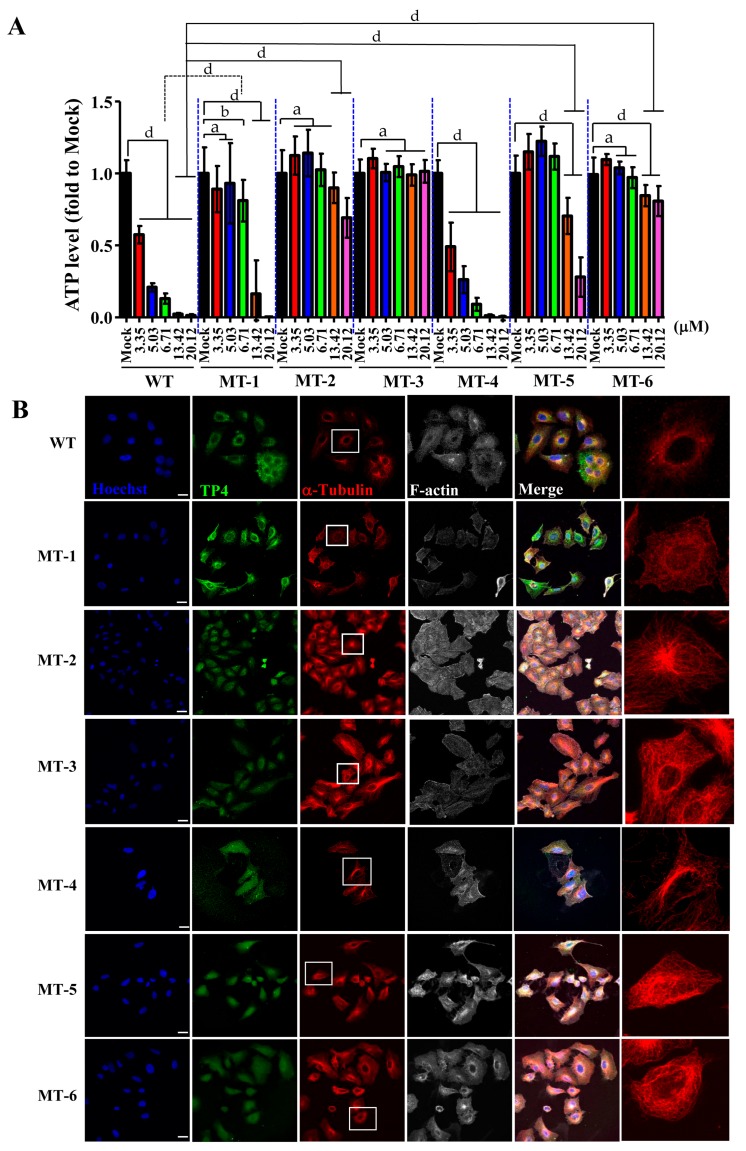
Loss of cancer-killing ability in TP4 mutants. (**A**) Cell viability in A549 cells was determined by the adenosine triphosphate (ATP) assay following treatment with varying doses of TP4 and TP4 mutants for 24 h. Multiple wells were analyzed for each assay. Results represent the mean ± SD. Statistical comparisons between groups were performed using two-way ANOVA: a, not significant; b, *p* < 0.05; d, *p* < 0.001. Statistical analyses are shown in Supplementary Table S1. (**B**) Cells were stained for TP4 (WT panel), FITC (MT-1 to MT-6 panels), α-Tubulin, and F-actin. Hoechst33342 was used for nuclei staining. Boxed regions are magnified in the furthest right panels. Note that microtubule abnormalities were observed in WT, MT-1, and MT-4-treated cells. Bar: 20 μm.

Because TP4 mutants showed a decreased suppression of microtubule polymerization, we next asked whether the mutations may also influence cancer cell killing activity. A549 cells were treated with different doses of TP4 variants, and the TP4-treated group showed obvious dose-dependent cytotoxic effects, see Figure 6A. Similar findings were observed with the Phe1 to Ala (MT-4) mutant, which was strongly cytotoxic to A549 cells, as shown in Figure 6A. Meanwhile, the Phe1 to Gly (MT-1) mutant showed decreased cytotoxicity, see Figure 6A. Notably, treatment with 6.71 μM MT-1 caused significantly less toxicity compared to wild-type TP4 (relative ATP levels: 0.852 ± 0.145 vs. 0.130 ± 0.036, *p* < 0.001), see Figure 6A and Supplementary Table S1. In MT-5 treated cells, no toxicity was observed at doses of 3.35–6.71 μM (relative ATP levels: 1.177 ± 0.125, 1.240 ± 0.102, and 1.122 ± 0.091 for the three doses). Moreover, MT-5-treated cells were significantly more viable than wild-type TP4-treated cells at doses 13.42 and 20.12 μM (relative ATP levels: 0.649 ± 0.126 and 0.226 ± 0.136 for MT-5-treated vs. 0.019 ± 0.009 and 0.010 ± 0.010 for wild-type TP4-treated, *p* < 0.001), see Figure 6A and Supplementary Table S1. Similar results were observed in MT-2 and MT-6 treated groups, with decreased cytotoxicity at high doses (13.42 and 20.12 μM) compared to the TP4-treated group (relative ATP levels: 0.895 ± 0.106 and 0.687 ± 0.139 for MT-2-treated group; 0.847 ± 0.074 and 0.808 ± 0.104 for MT-6-treated group; 0.019 ± 0.009 and 0.010 ± 0.010 for TP4-treated group, *p* < 0.001), see Figure 6A and Supplementary Table S1. No cytotoxicity was observed in MT-3-treated cells compared to vehicle, see Figure 6A and Supplementary Table S1. While the reduced toxicity may result from impaired binding to tubulin, it is also possible that such effects may be caused by conformational changes in the TP4 mutants, see Supplementary Figure S1C,D, which prevent intracellular penetration. To test this possibility, ICC was conducted to evaluate the cellular localization and the effects on microtubules. In TP4-treated cells (6.71 μM, 3 h), peptide penetration and microtubule defects were clearly observed, see Figure 6B, WT panel. For both Gly and Ala substitution mutants, FITC-labeled TP4 mutants were able to be taken up intracellularly, see Figure 6B, panels MT-1 to MT-6. However, the microtubule network was only affected in MT-1- and MT-4-treated cells and not the other mutant-treated groups, as shown in Figure 6B. These findings are consistent with the results from the cell viability assay, which showed that both MT-1 and MT-4 retain toxicity to the A549 cells, see Figure 6A.

## 3. Discussion

In this work, we found that the antimicrobial peptide, TP4, functions as a novel microtubule depolymerizing agent. Intracellular TP4 targets the microtubule network, where its actions potentially contribute to TP4-induced cancer cell death. The disruption of microtubules appears to require specific interactions between TP4 and α-Tubulin, as shown in Figure 3D and Figure 4D, at TP4 residues, Phe1 or Ile16 and Arg23. Substitution of Phe1, Ile16, and Arg23 by Gly or Ala decreases TP4-mediated microtubule depolymerization and cytotoxicity, as shown in Figure 5, indicating that microtubule targeting is a mechanistic requirement for TP4-mediated cytotoxicity.

Although intracellular penetration of AMPs has been reported to alter cytoskeletal structures, the mechanism linking AMP exposure to disruption of the cytoskeleton has not been previously reported [21,22]. The ixosin-B-amide-derived AMP, MAP-04-03, was shown to cause the collapse of cortical α-Tubulin and F-actin at a concentration of 25 μM [21], and CecropinXJ, an AMP derived from the silkworm (*Bombyx mori*), affects microtubule depolymerization and actin polymerization by attenuating the expression of the genes encoding α-actin, β-actin, γ-actin, α-Tubulin, and β-Tubulin [22]. Taking these studies together with our data, it is becoming apparent that microtubule disruption may be an important mechanism by which some AMPs kill cancer cells. Interference with microtubule dynamics has been a highly successful therapeutic modality in cancer. Several small molecule microtubule-targeting agents (MTAs), including both microtubule stabilizers and destabilizers, have been developed and are widely used to treat various cancer types. Independent of whether microtubules are stabilized or destabilized, disruption of microtubule dynamics is well known to cause deregulation of the microtubule network, leading to cell cycle arrest and cell death. For example, taxanes (e.g. paclitaxel and docetaxel) bind to the taxane-binding site within the β-subunit of microtubules. This binding enhances the affinity between tubulin monomers, increasing polymerization and protecting the filaments from disassembly [23,24]. Forced microtubule stabilization then impacts the configuration of the metaphase spindle, disrupting cell cycle progression, blocking mitosis, and triggering apoptosis [25,26]. On the other hand, vinca alkaloids and colchicine-associated compounds are microtubule destabilizing agents that bind the vinca and colchicine domains of tubulin, respectively. Vincas (e.g. Vincristine) interact with α- and β-Tubulin at the interface to form a spiral-like tubulin structure, blocking microtubule assembly [27]. In addition, the vinca domain on the β-subunit is near to the guanosine triphosphate (GTP)-binding site, and vinca binding inhibits GTP hydrolysis and guanosine diphosphate (GDP)/GTP exchange [28]. Vincas induce cell death by altering spindle microtubule dynamics to block mitosis (i.e, reducing mitotic spindle assembly) and inhibiting the transition from metaphase to anaphase [29,30]. Colchicine-associated compounds bind to the intra-dimeric α-β interface of tubulin heterodimers; thus, the binding site is located in a lumen instead of on an interacting surface. Binding to this colchicine domain leads to microtubule destabilization and disassembly through an intra-dimer bending formation, caused by disruption of the lateral interaction between tubulin molecules [31,32]. Among colchicine domain-binding drugs, Combretastatin family compounds are the most potent antineoplastic agents. These molecules function by inhibiting microtubule polymerization, preventing cell cycle progression at mitosis, and triggering apoptotic cell death [33,34]. In our study, we found that TP4 inhibits tubulin polymerization, see Figure 5C, indicating a function as a microtubule destabilizing agent. The Ile16 and Arg23 residues of the TP4 may play a critical role in suppressing microtubule polymerization, as evidenced by our mutation analysis. Ile16 and Arg23 of TP4 are expected to interact with Glu417 and His192/Asp424 in α-Tubulin, respectively, see Figure 4F,G. His192 is located in helix H5 and Glu417/Asp424 are located in helix H12 of the α-Tubulin molecule. These residues are conserved in all α-Tubulins across species and are required for zinc ion binding [35]. Importantly, the zinc ion participates in lateral interactions between protofilaments in zinc-induced tubulin polymers [35], suggesting that the binding of TP4 to α-Tubulin may disrupt lateral contact between protofilaments. In addition, TP4 Arg23 is expected to interact with α-Tubulin Leu195/Glu196, see Figure 4G. The Leu195 residue is conserved in α-Tubulin isotypes and is located in the external domain, which is required for the interaction of microtubule binding proteins [36,37]. Mutation of this residue was shown to affect microtubule stability in Taxol/EpoB-resistant EpoB480 cells [38]. The Glu196 residue is adjacent to Leu195, and it has been reported that mutations in Glu195, His197, and Asp199 in yeast are recessive lethal [39], indicating that these residues are critical for cell survival. Furthermore, the Ile16 (or Phe1) residue in TP4 is expected to interact with Tyr103 and His107 in α-Tubulin, see Figure 3F and Figure 4F. It has been reported that the organophosphorus agent, chlorpyrifos oxon, disrupts tubulin polymerization through binding to the Tyr103 residue in the EDAANNYR domain of α-Tubulin to cause neurotoxicity [40]. A recent report indicated that a de novo heterozygous mutation (320A>G) in the *TUB1A* gene, which encodes a His107 to Arg mutation in α-Tubulin, caused a wide spectrum of neurological problems, including lissencephaly [41]. Altogether, these findings support the notion that the TP4-Tubulin interaction may significantly affect microtubule maintenance in cancer cells to mediate TP4-induced toxicity to cancer cells.

Interestingly, AMPs have also been reported to target certain organelles, such as mitochondria or endoplasmic reticulum (ER), leading to the disruption of calcium homeostasis and induction of stress-responsive genes [11,13]. In fact, TP4 treatment was shown to activate the stress-induced transcription factor, FOSB, to trigger necrotic death in cancer cells [11]. Thus, TP4 induces a very different mechanism of cell death compared to other microtubule destabilizing agents, which commonly cause apoptotic cell death through mitotic suppression.

Overall, our study shows that a synthetic form of Nile tilapia-derived TP4 harbors microtubule destabilizing activity, and the essential residues required for the interaction of TP4 and Tubulin have been identified. These results are of particular importance for further development of AMP-based drugs, which may target the microtubule cytoskeleton for cancer treatment.

## 4. Material and Methods

### 4.1. Reagents and Peptide Sequence Analysis

TP4 (FIHHIIGGLFSAGKAIHRLIRRRRR), TP4 mutants, and Fluorescein isothiocyanate (FITC)-conjugated TP4 were synthesized and purified by GL Biochem Ltd. (Shanghai, China) as previously described [11]. Helical wheel projections of TP4 and TP4 mutants were plotted with RZ Lab (http://rzlab.ucr.edu/scripts/wheel/wheel.cgi). Structural models of TP4 and TP4 mutants were generated by using the PEP-FOLD 3.5 web server program (http://bioserv.rpbs.univ-paris-diderot.fr/services/PEP-FOLD3/) [42,43,44].

### 4.2. Protein-Peptide Docking

Homology modeling was conducted using “chain A” from the crystal structure of the tubulin-RB3-TTL-Zampanolide complex (PDB ID: 4I4T) as the template for human tubulin alpha-1C (UniProtKB Accession: Q9BQE3.1). MODELLER v9.17 (https://salilab.org/modeller/) [45] was utilized to predict the 3D structure of tubulin protein. The quality of the target models was validated with PROCHECK (https://www.ebi.ac.uk/thornton-srv/software/PROCHECK/) [46] and ProSA web (https://prosa.services.came.sbg.ac.at/prosa.php) [47]. Subsequent protein-peptide docking simulations were performed using the CABS-dock web server [48]. The TP4 peptide secondary structure was automatically defined using the PSIPRED (PSI-BLAST-based secondary structure prediction) method in CABS-dock. CABS-dock does not require a priori information about the binding site and allows complete peptide flexibility during the docking prediction. The docking results were analyzed according to clustering that was based on the root mean square deviation (RMSD) of the entire protein-peptide complex. Docked complexes were grouped in clusters according to similarity, and clusters were ranked according to size. Docked complexes were visualized and analyzed using the PyMOL Molecular Graphics System (Ver. 2.0 Schrödinger, Portland, OR, USA) and LigPlot^+^ 1.4.5 program (https://www.ebi.ac.uk/thornton-srv/software/LigPlus/) [49].

### 4.3. Cell Culture and Cell Viability Assay

The A549 cell line (Bioresource Collection and Research Center (BCRC) 60074) was purchased from the BCRC and cultured according to BCRC recommendations. For the cell viability and transfection assay, 4–5 × 10^3^ cells were seeded into the wells of a 96-well plate and cultured overnight. Cells were then treated according to the indicated doses and times. Cell viability was quantified using the CellTiter-Glo® Luminescent Cell Viability Assay kit (ATP assay) (Madison, WI, USA) as previously described [11]. The luminescent signal was measured using a photometer (SpectraMax® i3, Molecular Devices, Wals, Austria).

### 4.4. Co-Immunoprecipitation, LC-MS/MS Analysis, and Western Blot

Cell lysates were prepared in immunoprecipitation (IP) detergent (50 mM Tris-HCl, pH 8.0, 150 mM NaCl, 1% Igepal CA-630: Sigma-Aldrich, St. Louis, MO, USA) with protease cocktail (Roche Applied Science, Mannheim, Germany). For the IP assay, protein lysate (1 mg) from TP4-treated A549 cells (10 μg/mL, 3 h) was incubated with rabbit anti-TP4 antibody and magnetic beads (Dynabeads^TM^, Thermo Fisher Scientific, Oslo, Norway) in accordance with the recommended protocol. TP4 antibody was generated by MDBio, Inc (Taipei, Taiwan). Ovalbumin-conjugated TP4 synthetic peptide was injected into rabbits with Freund’s complete adjuvant (Sigma-Aldrich, Bangalore, India). The antisera were collected and processed for Western blotting (1:1000 dilution). For LC-MS/MS analysis, boiled lysates were electrophoresed on 15% or 6% SDS-PAGE, and gels were stained by InstantBlue™ solution (Expedeon Ltd, Cambridgeshire, UK). Excised protein slices were processed for tryptic in-gel digestion and LC-MS/MS analysis (Q-Exactive LC-MS, Thermo Scientific, Waltham, MA, USA). The mass spectrometry data were analyzed by Mascot engine (v.2.6.0) (Matrix Science Inc., Boston, MA, USA) to identify candidate interaction targets. For Western blots, samples were electrophoresed and transferred onto a Polyvinylidene difluoride (PVDF) membrane. The membranes were incubated in blocking buffer (0.1 M PBS, 5% non-fat milk, 0.2% Tween-20) for 1 hour at room temperature and then incubated in the same solution with primary or secondary antibodies (GE Healthcare Life Science, Buckinghamshire, UK). The primary antibodies used were as follows: α-Tubulin (1:5000, clone DM1A, Cell Signaling, Danvers, MA, USA) and GAPDH (1:10000, clone 6C5, EMD Millipore, Burlington, MA, USA). Signals were detected by enhanced chemiluminescence (Immobilon Western Chemiluminescent HRP substrate, Merck Millipore, Billerica, MA, USA) on an imaging system (UVP, BioSpectrumTM 500, Upland, CA, USA).

### 4.5. Immunocytochemical and Immunohistochemical Studies

Cells were stained with TP4 (1:1000), FITC (1:500, ThermoFisher Scientific, clone 1F8-1E4), α-Tubulin (1:500), and appropriate Alexa Flour conjugated secondary antibody (1:500; ThermoFisher Scientific, Eugene, OR, USA). Hochest33342 was used for nuclear staining, and Alexa Fluor 647-conjugated phalloidin (1:200, Thermo Fisher Scientific, Eugene, OR, USA) was used to stain F-actin. For the confocal microscopic analysis, samples were mounted with fluorescent mounting medium (ProLong Gold Antifade Reagent, ThermoFisher Scientific, Eugene, OR, USA) and images were obtained with a FV3000 laser-scanning confocal microscope (Olympus), using a 60 × objective lens (Plapon 60 × OSC2, N.A. 1.4, oil) with the 4′,6-diamidino-2-phenylindole (DAPI) (EX 461, EM 359), green fluorescent protein (GFP) (EX 470, EM 525 for enhanced GFP (EGFP)), and Cy5 (EX 670, EM 649) emission filters. FV31S software (Olympus, Tokyo, Japan) was used for image acquisition.

### 4.6. Microtubule Regrowth and In Vitro Tubulin Polymerization Assay

The Microtubule/Tubulin In Vivo Assay (Cytoskeleton, Inc., Denver, CO, USA) was performed using the “inhibitor condition” provided in the manufacturer’s instructions. Microtubule regrowth was assayed as described previously [50]. Briefly, cells with or without 3 h, 10 μg mL^−1^ TP4 treatment were incubated with nocodazole (20 μM) for 6 h at 37 °C. After washing with warm culture medium, cells were incubated for a further 0, 10, or 30 minutes. Fixed cells were stained with the α-Tubulin antibody and for F-actin. Confocal images were acquired on an FV3000 microscope (Olympus Corp., Tokyo, Japan).

### 4.7. Statistical analysis

For the multi-well based assay, cells were plated at least in quadruplicate. Data were collected from independently repeated experiments (*N* ≥ 3) and were analyzed by Prism5 software (GraphPad Inc., La Jolla, CA, USA). Statistical significance was determined by a two-tailed Student’s t-test or one-way/two-way analysis of variance (ANOVA) with a Bonferroni post hoc test. Differences were considered statistically significant at *p* < 0.05.

## Supplementary Materials

The following are available online at http://www.mdpi.com/1660-3397/16/12/462/s1. **Figure S1**: The α-helical and three-dimensional structures of Nile TP4 and its variants. (**A**–**G**) The helical projection plot and structural model of TP4 (**A**) and TP4 variants (MT-1 to MT-6, **B**–**G**). The helical projection was produced by an online helical wheel projection program (http://rzlab.ucr.edu/scripts/wheel/wheel.cgi). Amino acid residues are numbered and connected each amino acid with lines for presenting its relative position along the helix. The hydrophobic residues are shown as diamonds; hydrophilic residues are circles. Residues which are negatively charged are shown as triangles and those which are positively charged are shown as pentagons. Hydrophobicity is color coded. The most hydrophobic residue is shown in green; while the most hydrophilic residue is shown in red. Amino acids with zero hydrophobicity are shown in yellow. (**H**) Structural models of TP4 and its variants were generated by PEP-FOLD 3.5 program. **Table S1**: Cellular toxicity of TP4 to A549 cells was evaluated by cell viability. Statistical test results corresponding to Figure 6A are shown. Multiple wells were analyzed for each experiment (N ≥ 5 wells per dose). Results represent the mean ± SD from three independent assays. Statistical comparisons between mock versus TP4 treatment groups were performed using one-way ANOVA analysis with Bonferroni post-hoc test: a) not significant; b) *p* < 0.05; c) *p* < 0.01; d) *p* < 0.001. **Data S1**: α-Tubulin sequence identification and characterization through the Mascot database search.
