# Supplementary material for: Nile Tilapia Derived Antimicrobial Peptide TP4 Exerts Antineoplastic Activity Through Microtubule Disruption
Source: Mar Drugs. 2018 Nov 22;16(12):462. doi: 10.3390/md16120462 (PMC6315541; doi:10.3390/md16120462)
Supplement: Supplementary file 1 [file marinedrugs-16-00462-s001.pdf]

Supplementary files

Nile tilapia derived antimicrobial peptide TP4 exerts antineoplastic activity through microtubule disruption

Chen-Hung Ting[1], Yi-Chung Liu[2], Ping-Chiang Lyu[3], Jyh-Yih Chen[1]*

1. Marine Research Station, Institute of Cellular and Organismic Biology, Academia Sinica, 23-10 Dahuen Rd., Jiaushi, Ilan 262, Taiwan.
2. Institute of Population Sciences, National Health Research Institutes, 35 Keyan Rd., Zhunan, Miaoli County 350, Taiwan.
3. Institute of Bioinformatics and Structural Biology, National Tsing-Hua University, 101, Sec. 2, Kuang-Fu Rd., Hsinchu 300, Taiwan.

*correspondence：

E-mail: zoocjy@gate.sinica.edu.tw; Tel.: 886-920802111; Fax: 886-39871035

Running title: TP4 destabilizes microtubules in cancer cells

Treatment	Mock	3.35	5.03	6.71	13.42	20.12 (μM)
WT	0.985±0.091	0.580±0.061^{d}	0.211±0.030^{d}	0.129±0.034^{d}	0.020±0.009^{d}	0.009±0.010^{d}
MT-1	0.952±0.182	0.931±0.160^{a}	1.044±0.280^{a}	0.852±0.145^{b}	0.007±0.233^{d}	0.003±0.001^{d}
MT-2	1.045±0.161	1.146±0.133^{a}	1.089±0.162^{b}	0.974±0.113^{a}	0.895±0.106^{a}	0.687±0.139^{d}
MT-3	0.996±0.097	1.126±0.068^{b}	0.999±0.060^{a}	1.043±0.073^{a}	0.980±0.075^{a}	1.027±0.076^{a}
MT-4	0.985±0.091	0.454±0.169^{d}	0.241±0.094^{d}	0.070±0.045^{d}	0.007±0.009^{d}	0.001±0.007^{d}
MT-5	1.025±0.125	1.177±0.125^{c}	1.240±0.102^{d}	1.122±0.09^{a}	0.649±0.126^{d}	0.226±0.136^{d}
MT-6	1.003±0.118	1.097±0.039^{c}	1.034±0.044^{a}	0.971±0.072^{a}	0.847±0.074^{d}	0.808±0.104^{d}

Supplementary Table S1. Cellular toxicity of TP4 to A549 cells was evaluated by cell viability. Results of statistical tests on data in Figure 6A are shown. Multiple wells were analyzed for each experiment (n ≥ 5 wells per dose). Results represent the mean ± SD from three independent assays. Statistical comparisons between mock versus TP4 treatment groups were performed using two-way ANOVA analysis with Bonferroni post-hoc test: a, not significant; b, $P < 0.05$; c, $P < 0.01$; d, $P < 0.001$.

Supplementary Figure legends

Supplementary Figure S1. The α-helical and three-dimensional structures of TP4 and its variants. (A-G) The helical projection plot and structural model of TP4 (A) and TP4 variants (MT-1 to MT-6, B-G). The helical projection was produced by an online helical wheel projection program (http://rzlab.ucr.edu/scripts/wheel/wheel.cgi). Amino acid residues are numbered and each amino acid is connected with lines to indicate its relative position along the helix. The hydrophobic residues are shown as diamonds; hydrophilic residues are circles. Residues with negative charge are shown as triangles and positively charged residues are pentagons. Hydrophobicity is color coded. The

most hydrophobic residue is shown in green; while the most hydrophilic residue is shown in red. Amino acids with zero hydrophobicity are shown in yellow. (H) Structural models of TP4 and its variants were generated by PEP-FOLD 3.5 program (http://mobyle.rpbs.univ-paris-diderot.fr/cgi-bin/portal.py#forms::PEP-FOLD).

Supplementary Dataset S1. α-Tubulin sequence identification and characterization using a Mascot database search.

Supplementary Figure S1

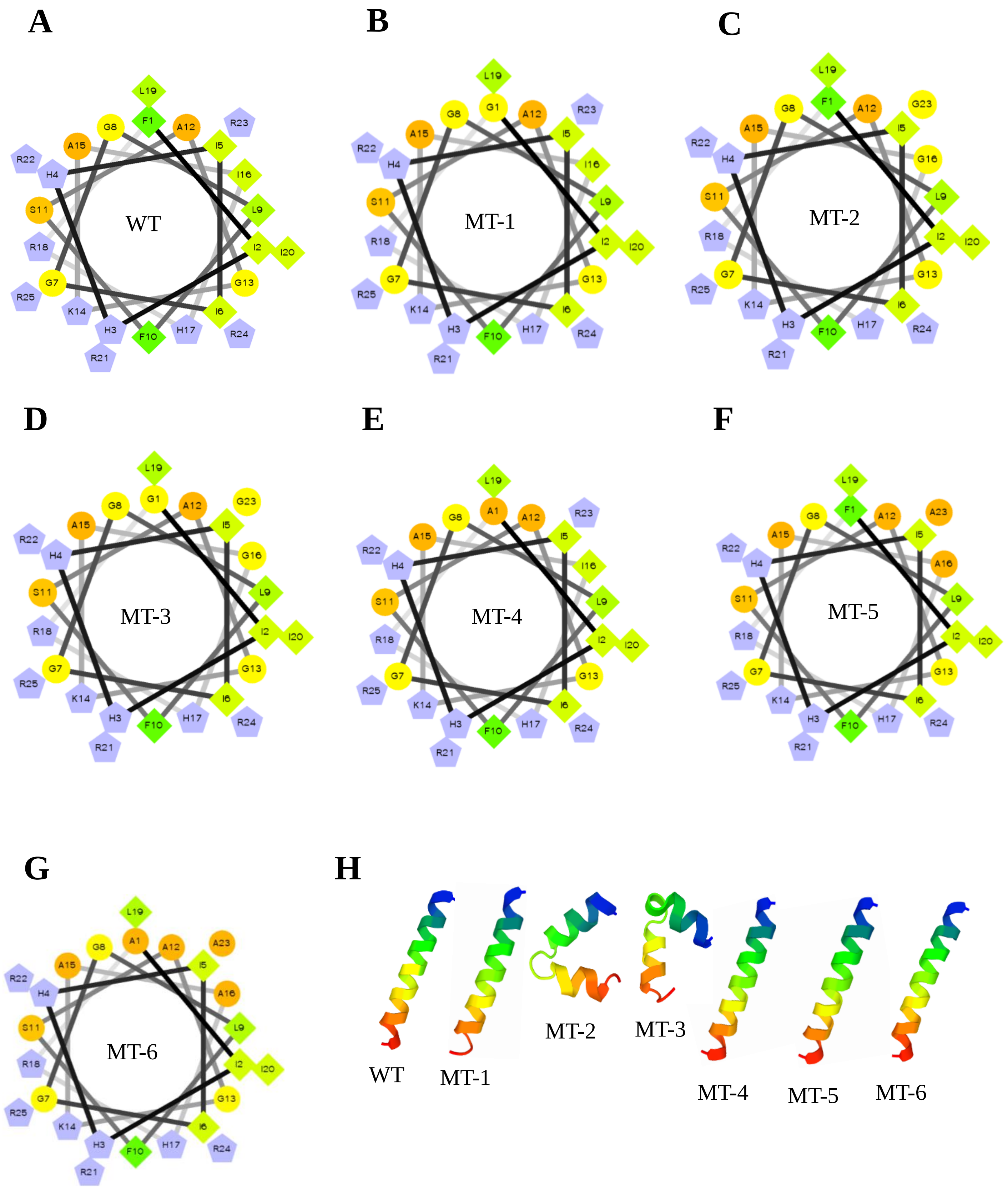

MATRIX SCIENCE MASCOT Search Results

Protein View: TBA1B_HUMAN

Tubulin alpha-1B chain OS=Homo sapiens GN=TUBA1B PE=1 SV=1

**Database:**	SwissProt
**Score:**	1436
**Monoisotopic mass (M_r):**	50804
**Calculated pI:**	4.94
**Taxonomy:**	Homo sapiens

Sequence similarity is available as an NCBI BLAST search of TBA1B_HUMAN against nr.

Search parameters

**MS data file:**	A2018042402.mgf
**Enzyme:**	Trypsin: cuts C-term side of KR unless next residue is P.
**Fixed modifications:**	Carbamidomethyl (C)
**Variable modifications:**	Deamidated (NQ), Oxidation (M)

Protein sequence coverage: 47%

Matched peptides shown in ***bold red***.

1 MRECISIHVG QAGVQIGNAC WELYCLEHGI QPDGQMPSDK **TIGGGDDSFN**
51 **TFFSETGAGK HVPRAVFVDL EPTVIDEVRT GTYRQLFHPE QLITGKEDAA**
101 **NNYAR**GHYTI GKEIIDLVLD RIRKLADQCT GLQGFLVFHS FGGGTGSGFT
151 SLLMER**LSVD YGKK**SKLEFS IYPAPQVSTA VVEPYNSILT THTTLEHSDC
201 AFMVDNEAIY DICRR**NLDIE RPTYTNLNRL ISQIVSSITA SLR**FDGALNV
251 DLTEFQTNLV PYPR**IHFPLA TYAPVISAEK AYHEQLSVAE ITNACFEPAN**
301 **QMVK**CDPRHG K**YMACCLLYR GDVVPKDVNA AIATIK**TKRS IQFVDWCPTG
351 FK**VGINYQPP TVVPGGDLAK** VQR**AVCMLSN TTAIAEAWAR LDHKFDLMYA**
401 **K**RAFVHWYVG EGMEEGEFSE AREDMAALEK DYEEVGVDSV EGEGEEEGEE
451 Y

Unformatted sequence string: 451 residues (for pasting into other applications).

Sort by (●) residue number ( ) increasing mass ( ) decreasing mass
Show (●) matched peptides only ( ) predicted peptides also

Query	Start – End	Observed	Mr(expt)	Mr(calc)	ppm	M	Score	Expect	Rank	U	Peptide
18754	41 – 60	1004.4420	2006.8694	2006.8858	-8.19	0	78	1.1e-007	1		K.TIGGGDDSFNTFFSETGAGK.H
18755	41 – 60	1004.4484	2006.8823	2006.8858	-1.74	0	72	5e-007	1		K.TIGGGDDSFNTFFSETGAGK.H
20563	41 – 64	625.0458	2496.1540	2496.1670	-5.20	1	60	1.4e-005	1		K.TIGGGDDSFNTFFSETGAGKHVPR.A
20564	41 – 64	625.0465	2496.1567	2496.1670	-4.13	1	45	0.00046	1		K.TIGGGDDSFNTFFSETGAGKHVPR.A
20565	41 – 64	833.3925	2497.1557	2497.1510	1.89	1	90	1.5e-008	1		K.TIGGGDDSFNTFFSETGAGKHVPR.A + Deamidate
17274	65 – 79	851.4505	1700.8864	1700.8985	-7.09	0	73	6.9e-007	1		R.AVFVDLEPTVIDEVR.T
17275	65 – 79	567.9715	1700.8927	1700.8985	-3.43	0	61	1.1e-005	1		R.AVFVDLEPTVIDEVR.T
17276	65 – 79	851.4553	1700.8960	1700.8985	-1.50	0	79	1.4e-007	1		R.AVFVDLEPTVIDEVR.T
19695	65 – 84	760.7340	2279.1800	2279.1798	0.11	1	39	0.0016	1		R.AVFVDLEPTVIDEVRTGTYR.Q
20228	85 – 105	604.5551	2414.1913	2414.1978	-2.69	1	70	1.4e-006	1		R.QLFHPEQLITGKEDAANNYAR.G
20229	85 – 105	604.5554	2414.1923	2414.1978	-2.28	1	59	1.8e-005	1		R.QLFHPEQLITGKEDAANNYAR.G
20232	85 – 105	604.8018	2415.1782	2415.1818	-1.52	1	73	7.4e-007	1		R.QLFHPEQLITGKEDAANNYAR.G + Deamidated (
20233	85 – 105	806.0674	2415.1805	2415.1818	-0.56	1	66	4.1e-006	1		R.QLFHPEQLITGKEDAANNYAR.G + Deamidated (
20234	85 – 105	604.8028	2415.1821	2415.1818	0.11	1	68	2.2e-006	1		R.QLFHPEQLITGKEDAANNYAR.G + Deamidated (
20235	85 – 105	806.0704	2415.1895	2415.1818	3.16	1	71	1.1e-006	1		R.QLFHPEQLITGKEDAANNYAR.G + Deamidated (
20237	85 – 105	806.3966	2416.1680	2416.1658	0.89	1	56	4.5e-005	1		R.QLFHPEQLITGKEDAANNYAR.G + 2 Deamidated
20238	85 – 105	605.0531	2416.1833	2416.1658	7.22	1	37	0.0028	1		R.QLFHPEQLITGKEDAANNYAR.G + 2 Deamidated
10627	157 – 163	391.2082	780.4018	780.4018	0.0051	0	38	0.0015	1		R.LSVDYGK.K
11398	157 – 164	455.2555	908.4964	908.4967	-0.39	1	36	0.0017	1		R.LSVDYGKK.S
11399	157 – 164	455.2555	908.4964	908.4967	-0.32	1	32	0.0041	1		R.LSVDYGKK.S
17339	216 – 229	859.9405	1717.8664	1717.8747	-4.82	0	54	6.3e-005	1		R.NLDIERPTYTNLNR.L
15844	230 – 243	744.4402	1486.8658	1486.8719	-4.06	0	75	9.3e-008	1		R.LISQIVSSITASLR.F
15845	230 – 243	496.6305	1486.8697	1486.8719	-1.44	0	52	1.6e-005	1		R.LISQIVSSITASLR.F
17513	265 – 280	586.3240	1755.9503	1755.9559	-3.21	0	49	0.00011	1		R.IHFPLATYAPVISAEK.A
17514	265 – 280	586.3248	1755.9527	1755.9559	-1.86	0	53	3.6e-005	1		R.IHFPLATYAPVISAEK.A
21364	281 – 304	917.4270	2749.2592	2749.2840	-9.01	0	40	0.0012	1		K.AYHEQLSVAEITNACFEPANQMVK.C
14315	312 – 320	625.2797	1248.5449	1248.5453	-0.36	0	45	0.00023	1		K.YMACCLLYR.G
16516	321 – 336	805.9562	1609.8979	1609.9039	-3.72	1	53	2.6e-005	1		R.GDVVPKDVNAAIATIK.T
12324	327 – 336	508.2937	1014.5728	1014.5709	1.88	0	32	0.011	1		K.DVNAAIATIK.T
17908	353 – 370	912.9936	1823.9726	1823.9782	-3.04	0	40	0.00079	1		K.VGINYQPPTVVPGGDLAK.V
18189	374 – 390	932.9509	1863.8872	1863.8971	-5.32	0	102	1.1e-009	1		R.AVCMLSNTTAIAEAWAR.L
18190	374 – 390	622.3030	1863.8873	1863.8971	-5.27	0	87	3.1e-008	1		R.AVCMLSNTTAIAEAWAR.L
18197	374 – 390	933.4531	1864.8916	1864.8811	5.60	0	69	2.1e-006	1		R.AVCMLSNTTAIAEAWAR.L + Deamidated (NQ)
18311	374 – 390	627.6362	1879.8869	1879.8920	-2.75	0	31	0.014	1		R.AVCMLSNTTAIAEAWAR.L + Oxidation (M)
15279	391 – 401	466.2349	1395.6828	1395.6857	-2.08	1	31	0.011	1		R.LDHKFDLMYAK.R + Oxidation (M)

Error: try setting browser cache to automatic.

ID TBA1B_HUMAN Reviewed; 451 AA.
AC P68363; P04687; P05209; Q27I68; Q8WU19;

```
DT   13-AUG-1987, integrated into UniProtKB/Swiss-Prot.
DT   13-AUG-1987, sequence version 1.
DT   15-MAR-2017, entry version 143.
DE   RecName: Full=Tubulin alpha-1B chain;
DE   AltName: Full=Alpha-tubulin ubiquitous;
DE   AltName: Full=Tubulin K-alpha-1;
DE   AltName: Full=Tubulin alpha-ubiquitous chain;
DE   Contains:
DE     RecName: Full=Detyrosinated tubulin alpha-1B chain;
GN   Name=TUBA1B;
OS   Homo sapiens (Human).
OC   Eukaryota; Metazoa; Chordata; Craniata; Vertebrata; Euteleostomi;
OC   Mammalia; Eutheria; Euarchontoglires; Primates; Haplorrhini;
OC   Catarrhini; Hominidae; Homo.
OX   NCBI_TaxID=9606;
RN   [1]
RP   NUCLEOTIDE SEQUENCE [MRNA] (ISOFORM 1).
RC   TISSUE=Keratinocyte;
RX   PubMed=6646120; DOI=10.1128/MCB.3.10.1738;
RA   Cowan N.J., Dobner P., Fuchs E.V., Cleveland D.W.;
RT   "Expression of human alpha-tubulin genes: interspecies conservation of
RT   3' untranslated regions.";
RL   Mol. Cell. Biol. 3:1738-1745(1983).
RN   [2]
RP   NUCLEOTIDE SEQUENCE [MRNA] (ISOFORM 1).
RA   de Hostos E.L.;
RL   Submitted (JUL-1998) to the EMBL/GenBank/DDBJ databases.
RN   [3]
RP   NUCLEOTIDE SEQUENCE [MRNA] (ISOFORM 1).
RA   Li Q., Liu L., Ma S.;
RT   "Gene response of gangliocytes stimulated by Herpes simplex virus type
RT   1.";
RL   Submitted (FEB-2006) to the EMBL/GenBank/DDBJ databases.
RN   [4]
RP   NUCLEOTIDE SEQUENCE [LARGE SCALE MRNA] (ISOFORMS 1 AND 2).
RC   TISSUE=Brain, Colon, Eye, Kidney, Lung, Muscle, Placenta, Prostate,
RC   Skin, and Uterus;
RX   PubMed=15489334; DOI=10.1101/gr.2596504;
RG   The MGC Project Team;
RT   "The status, quality, and expansion of the NIH full-length cDNA
RT   project: the Mammalian Gene Collection (MGC).";
RL   Genome Res. 14:2121-2127(2004).
RN   [5]
RP   PROTEIN SEQUENCE OF 41-60; 65-79; 113-121; 230-280; 312-320; 327-336;
RP   340-370; 374-390; 395-401 AND 403-422, AND IDENTIFICATION BY MASS
RP   SPECTROMETRY.
RC   TISSUE=Brain, Cajal-Retzius cell, and Fetal brain cortex;
RA   Lubec G., Afjehi-Sadat L., Vishwanath V., Chen W.-Q., Sun Y.;
RL   Submitted (DEC-2008) to UniProtKB.
RN   [6]
RP   PROTEIN SEQUENCE OF 41-79; 65-79; 85-121; 125-164; 216-304; 312-370
RP   AND 374-451, PHOSPHORYLATION AT SER-48 AND SER-232, METHYLATION AT
RP   ARG-339, AND IDENTIFICATION BY MASS SPECTROMETRY.
RC   TISSUE=Embryonic kidney;
RA   Bienvenut W.V., Waridel P., Quadroni M.;
RL   Submitted (MAR-2009) to UniProtKB.
RN   [7]
RP   PROTEIN SEQUENCE OF 353-370 AND 395-401.
RC   TISSUE=Brain;
RX   PubMed=8619814; DOI=10.1006/bbrc.1996.0211;
RA   Baumann M.H., Wisniewski T., Levy E., Plant G.T., Ghiso J.;
RT   "C-terminal fragments of alpha- and beta-tubulin form amyloid fibrils
RT   in vitro and associate with amyloid deposits of familial cerebral
RT   amyloid angiopathy, British type.";
RL   Biochem. Biophys. Res. Commun. 219:238-242(1996).
RN   [8]
RP   PROTEIN SEQUENCE OF 439-451, AND NITRATION AT TYR-451.
RX   PubMed=10339593; DOI=10.1073/pnas.96.11.6365;
RA   Eiserich J.P., Estevez A.G., Bamberg T.V., Ye Y.Z., Chumley P.H.,
RA   Beckman J.S., Freeman B.A.;
RT   "Microtubule dysfunction by posttranslational nitrotyrosination of
RT   alpha-tubulin: a nitric oxide-dependent mechanism of cellular
RT   injury.";
RL   Proc. Natl. Acad. Sci. U.S.A. 96:6365-6370(1999).
RN   [9]
RP   GLYCYLATION.
RX   PubMed=19524510; DOI=10.1016/j.cell.2009.05.020;
RA   Rogowski K., Juge F., van Dijk J., Wloga D., Strub J.-M.,
RA   Levilliers N., Thomas D., Bre M.-H., Van Dorsselaer A., Gaertig J.,
RA   Janke C.;
RT   "Evolutionary divergence of enzymatic mechanisms for posttranslational
RT   polyglycylation.";
RL   Cell 137:1076-1087(2009).
RN   [10]
RP   ACETYLATION AT LYS-40.
RX   PubMed=24906155; DOI=10.1016/j.cell.2014.03.061;
RA   Szyk A., Deaconescu A.M., Spector J., Goodman B., Valenstein M.L.,
RA   Ziolkowska N.E., Kormendi V., Grigorieff N., Roll-Mecak A.;
RT   "Molecular basis for age-dependent microtubule acetylation by tubulin
RT   acetyltransferase.";
RL   Cell 157:1405-1415(2014).
RN   [11]
RP   IDENTIFICATION BY MASS SPECTROMETRY [LARGE SCALE ANALYSIS].
RX   PubMed=25944712; DOI=10.1002/pmic.201400617;
RA   Vaca Jacome A.S., Rabilloud T., Schaeffer-Reiss C., Rompais M.,
RA   Ayoub D., Lane L., Bairoch A., Van Dorsselaer A., Carapito C.;
RT   "N-terminome analysis of the human mitochondrial proteome.";
RL   Proteomics 15:2519-2524(2015).
RN   [12]
RP   DETYROSINATION.
RX   PubMed=25908662; DOI=10.1126/science.aaa5175;
RA   Barisic M., Silva e Sousa R., Tripathy S.K., Magiera M.M.,
RA   Zaytsev A.V., Pereira A.L., Janke C., Grishchuk E.L., Maiato H.;
RT   "Mitosis. Microtubule detyrosination guides chromosomes during
RT   mitosis.";
RL   Science 348:799-803(2015).
RN   [13]
RP   GLUTAMYLATION.
RX   PubMed=26875866; DOI=10.1016/j.cell.2016.01.019;
```

```
RA   Valenstein M.L., Roll-Mecak A.;
RT   "Graded control of microtubule severing by tubulin glutamylation.";
RL   Cell 164:911-921(2016).
RN   [14]
RP   METHYLATION AT LYS-40.
RX   PubMed=27518565; DOI=10.1016/j.cell.2016.07.005;
RA   Park I.Y., Powell R.T., Tripathi D.N., Dere R., Ho T.H., Blasius T.L.,
RA   Chiang Y.C., Davis I.J., Fahey C.C., Hacker K.E., Verhey K.J.,
RA   Bedford M.T., Jonasch E., Rathmell W.K., Walker C.L.;
RT   "Dual chromatin and cytoskeletal remodeling by SETD2.";
RL   Cell 166:950-962(2016).
RN   [15]
RP   TYROSINATION.
RX   PubMed=26972003; DOI=10.1016/j.celrep.2016.02.046;
RA   Nirschl J.J., Magiera M.M., Lazarus J.E., Janke C., Holzbaur E.L.;
RT   "Alpha-tubulin tyrosination and CLIP-170 phosphorylation regulate the
RT   initiation of dynein-driven transport in neurons.";
RL   Cell Rep. 14:2637-2652(2016).
RN   [16]
RP   STRUCTURE BY NMR OF 416-451 IN COMPLEX WITH CLIP1.
RX   PubMed=17563362; DOI=10.1073/pnas.0703876104;
RA   Mishima M., Maesaki R., Kasa M., Watanabe T., Fukata M., Kaibuchi K.,
RA   Hakoshima T.;
RT   "Structural basis for tubulin recognition by cytoplasmic linker
RT   protein 170 and its autoinhibition.";
RL   Proc. Natl. Acad. Sci. U.S.A. 104:10346-10351(2007).
CC   -!- FUNCTION: Tubulin is the major constituent of microtubules. It
CC       binds two moles of GTP, one at an exchangeable site on the beta
CC       chain and one at a non-exchangeable site on the alpha chain.
CC   -!- SUBUNIT: Dimer of alpha and beta chains. A typical microtubule is
CC       a hollow water-filled tube with an outer diameter of 25 nm and an
CC       inner diameter of 15 nM. Alpha-beta heterodimers associate head-
CC       to-tail to form protofilaments running lengthwise along the
CC       microtubule wall with the beta-tubulin subunit facing the
CC       microtubule plus end conferring a structural polarity.
CC       Microtubules usually have 13 protofilaments but different
CC       protofilament numbers can be found in some organisms and
CC       specialized cells. {ECO:0000269|PubMed:17563362}.
CC   -!- INTERACTION:
CC       Q6RW13:AGTRAP; NbExp=3; IntAct=EBI-487083, EBI-741181;
CC       Q8AZK7:EBNA-LP (xeno); NbExp=3; IntAct=EBI-487083, EBI-1185167;
CC       Q13509:TUBB3; NbExp=3; IntAct=EBI-487083, EBI-350989;
CC   -!- SUBCELLULAR LOCATION: Cytoplasm, cytoskeleton.
CC   -!- ALTERNATIVE PRODUCTS:
CC       Event=Alternative splicing; Named isoforms=2;
CC       Name=1;
CC         IsoId=P68363-1; Sequence=Displayed;
CC       Name=2;
CC         IsoId=P68363-2; Sequence=VSP_055764;
CC         Note=No experimental confirmation available.;
CC   -!- PTM: Some glutamate residues at the C-terminus are
CC       polyglutamylated, resulting in polyglutamate chains on the gamma-
CC       carboxyl group (PubMed:26875866). Polyglutamylation plays a key
CC       role in microtubule severing by spastin (SPAST). SPAST
CC       preferentially recognizes and acts on microtubules decorated with
CC       short polyglutamate tails: severing activity by SPAST increases as
CC       the number of glutamates per tubulin rises from one to eight, but
CC       decreases beyond this glutamylation threshold (PubMed:26875866).
CC       {ECO:0000269|PubMed:26875866}.
CC   -!- PTM: Some glutamate residues at the C-terminus are monoglycylated
CC       but not polyglycylated due to the absence of functional TTLL10 in
CC       human. Monoglycylation is mainly limited to tubulin incorporated
CC       into axonemes (cilia and flagella). Both polyglutamylation and
CC       monoglycylation can coexist on the same protein on adjacent
CC       residues, and lowering glycylation levels increases
CC       polyglutamylation, and reciprocally. The precise function of
CC       monoglycylation is still unclear (Probable).
CC       {ECO:0000305|PubMed:19524510}.
CC   -!- PTM: Acetylation of alpha chains at Lys-40 is located inside the
CC       microtubule lumen. This modification has been correlated with
CC       increased microtubule stability, intracellular transport and
CC       ciliary assembly. {ECO:0000269|PubMed:24906155}.
CC   -!- PTM: Methylation of alpha chains at Lys-40 is found in mitotic
CC       microtubules and is required for normal mitosis and cytokinesis
CC       contributing to genomic stability. {ECO:0000305|PubMed:27518565}.
CC   -!- PTM: Nitration of Tyr-451 is irreversible and interferes with
CC       normal dynein intracellular distribution.
CC       {ECO:0000269|PubMed:10339593}.
CC   -!- PTM: Undergoes a tyrosination/detyrosination cycle, the cyclic
CC       removal and re-addition of a C-terminal tyrosine residue by the
CC       enzymes tubulin tyrosine carboxypeptidase (TTCP) and tubulin
CC       tyrosine ligase (TTL), respectively. {ECO:0000269|PubMed:25908662,
CC       ECO:0000269|PubMed:26972003}.
CC   -!- PTM: Tubulin alpha-1B chain: Tyrosination promotes microtubule
CC       interaction with CAP-Gly domain-containing proteins such as CLIP1,
CC       CLIP2 and DCTN1 (By similarity). Tyrosination regulates the
CC       initiation of dynein-dynactin motility via interaction with DCTN1,
CC       which brings the dynein-dynactin complex into contact with
CC       microtubules (PubMed:26972003). In neurons, tyrosinated tubulins
CC       mediate the initiation of retrograde vesicle transport (By
CC       similarity). {ECO:0000250|UniProtKB:P05213,
CC       ECO:0000250|UniProtKB:Q71U36, ECO:0000269|PubMed:26972003}.
CC   -!- PTM: Detyrosinated tubulin alpha-1B chain: Detyrosination is
CC       involved in metaphase plate congression by guiding chromosomes
CC       during mitosis: detyrosination promotes interaction with CENPE,
CC       promoting pole-proximal transport of chromosomes toward the
CC       equator (PubMed:25908662). Detyrosination increases microtubules-
CC       dependent mechanotransduction in dystrophic cardiac and skeletal
CC       muscle. In cardiomyocytes, detyrosinated microtubules are required
CC       to resist to contractile compression during contraction:
CC       detyrosination promotes association with desmin (DES) at force-
CC       generating sarcomeres, leading to buckled microtubules and
CC       mechanical resistance to contraction (By similarity).
CC       {ECO:0000250|UniProtKB:P05213, ECO:0000269|PubMed:25908662}.
CC   -!- SIMILARITY: Belongs to the tubulin family. {ECO:0000305}.
DR   EMBL; K00558; AAA91576.1; -; mRNA.
DR   EMBL; AF081484; AAC31959.1; -; mRNA.
DR   EMBL; DQ400107; ABD60581.1; -; mRNA.
DR   EMBL; BC000696; AAH00696.1; -; mRNA.
```

```
DR   EMBL; BC001128; AAH01128.1; -; mRNA.
DR   EMBL; BC006379; AAH06379.1; -; mRNA.
DR   EMBL; BC006481; AAH06481.1; -; mRNA.
DR   EMBL; BC008659; AAH08659.1; -; mRNA.
DR   EMBL; BC009314; AAH09314.1; -; mRNA.
DR   EMBL; BC009509; AAH09509.1; -; mRNA.
DR   EMBL; BC009512; AAH09512.1; -; mRNA.
DR   EMBL; BC009513; AAH09513.1; -; mRNA.
DR   EMBL; BC010494; AAH10494.1; -; mRNA.
DR   EMBL; BC011572; AAH11572.1; -; mRNA.
DR   EMBL; BC015883; AAH15883.1; -; mRNA.
DR   EMBL; BC017004; AAH17004.1; -; mRNA.
DR   EMBL; BC021564; AAH21564.1; -; mRNA.
DR   EMBL; BC030820; AAH30820.1; -; mRNA.
DR   EMBL; BC071904; AAH71904.1; -; mRNA.
DR   CCDS; CCDS31792.1; -. [P68363-1]
DR   PIR; I77403; I77403.
DR   RefSeq; NP_006073.2; NM_006082.2. [P68363-1]
DR   UniGene; Hs.524390; -.
DR   PDB; 2E4H; NMR; -; B=416-451.
DR   PDB; 5IJ0; EM; 3.80 A; A=1-437.
DR   PDB; 5IJ9; EM; 3.70 A; A=1-437.
DR   PDBsum; 2E4H; -.
DR   PDBsum; 5IJ0; -.
DR   PDBsum; 5IJ9; -.
DR   ProteinModelPortal; P68363; -.
DR   SMR; P68363; -.
DR   BioGrid; 115651; 124.
DR   IntAct; P68363; 40.
DR   MINT; MINT-4998849; -.
DR   STRING; 9606.ENSP00000336799; -.
DR   ChEMBL; CHEMBL2095182; -.
DR   DrugBank; DB05147; CYT997.
DR   DrugBank; DB03010; Epothilone B.
DR   DrugBank; DB01873; Epothilone D.
DR   iPTMnet; P68363; -.
DR   PhosphoSitePlus; P68363; -.
DR   SwissPalm; P68363; -.
DR   BioMuta; TUBA1B; -.
DR   DMDM; 55977474; -.
DR   OGP; P68363; -.
DR   SWISS-2DPAGE; P68363; -.
DR   EPD; P68363; -.
DR   MaxQB; P68363; -.
DR   PaxDb; P68363; -.
DR   PeptideAtlas; P68363; -.
DR   PRIDE; P68363; -.
DR   TopDownProteomics; P68363-1; -. [P68363-1]
DR   DNASU; 10376; -.
DR   Ensembl; ENST00000336023; ENSP00000336799; ENSG00000123416. [P68363-1]
DR   GeneID; 10376; -.
DR   KEGG; hsa:10376; -.
DR   UCSC; uc001rtm.4; human. [P68363-1]
DR   CTD; 10376; -.
DR   DisGeNET; 10376; -.
DR   GeneCards; TUBA1B; -.
DR   H-InvDB; HIX0079488; -.
DR   HGNC; HGNC:18809; TUBA1B.
DR   HPA; CAB011513; -.
DR   HPA; HPA039247; -.
DR   HPA; HPA043684; -.
DR   HPA; HPA063394; -.
DR   MIM; 602530; gene.
DR   neXtProt; NX_P68363; -.
DR   OpenTargets; ENSG00000123416; -.
DR   PharmGKB; PA162407332; -.
DR   eggNOG; KOG1376; Eukaryota.
DR   eggNOG; COG5023; LUCA.
DR   GeneTree; ENSGT00870000136497; -.
DR   HOGENOM; HOG000165711; -.
DR   HOVERGEN; HBG000089; -.
DR   InParanoid; P68363; -.
DR   KO; K07374; -.
DR   OMA; WARTRNT; -.
DR   OrthoDB; EOG091G0736; -.
DR   PhylomeDB; P68363; -.
DR   TreeFam; TF300314; -.
DR   Reactome; R-HSA-1445148; Translocation of GLUT4 to the plasma membrane.
DR   Reactome; R-HSA-190840; Microtubule-dependent trafficking of connexons from Golgi to the plasma membrane.
DR   Reactome; R-HSA-190861; Gap junction assembly.
DR   Reactome; R-HSA-2132295; MHC class II antigen presentation.
DR   Reactome; R-HSA-2467813; Separation of Sister Chromatids.
DR   Reactome; R-HSA-2500257; Resolution of Sister Chromatid Cohesion.
DR   Reactome; R-HSA-380320; Recruitment of NuMA to mitotic centrosomes.
DR   Reactome; R-HSA-389960; Formation of tubulin folding intermediates by CCT/TriC.
DR   Reactome; R-HSA-389977; Post-chaperonin tubulin folding pathway.
DR   Reactome; R-HSA-437239; Recycling pathway of L1.
DR   Reactome; R-HSA-5610787; Hedgehog 'off' state.
DR   Reactome; R-HSA-5617833; Cilium Assembly.
DR   Reactome; R-HSA-5620924; Intraflagellar transport.
DR   Reactome; R-HSA-5626467; RHO GTPases activate IQGAPs.
DR   Reactome; R-HSA-5663220; RHO GTPases Activate Formins.
DR   Reactome; R-HSA-6807878; COPI-mediated anterograde transport.
DR   Reactome; R-HSA-6811434; COPI-dependent Golgi-to-ER retrograde traffic.
DR   Reactome; R-HSA-6811436; COPI-independent Golgi-to-ER retrograde traffic.
DR   Reactome; R-HSA-68877; Mitotic Prometaphase.
DR   Reactome; R-HSA-8852276; The role of GTSE1 in G2/M progression after G2 checkpoint.
DR   Reactome; R-HSA-983189; Kinesins.
DR   SIGNOR; P68363; -.
DR   ChiTaRS; TUBA1B; human.
DR   EvolutionaryTrace; P68363; -.
DR   GeneWiki; TUBA1B; -.
DR   GenomeRNAi; 10376; -.
DR   PRO; PR:P68363; -.
DR   Proteomes; UP000005640; Chromosome 12.
DR   Bgee; ENSG00000123416; -.
DR   CleanEx; HS_TUBA1B; -.
DR   ExpressionAtlas; P68363; baseline and differential.
DR   Genevisible; P68363; HS.
```

```
DR   GO; GO:0005881; C:cytoplasmic microtubule; IEA:Ensembl.
DR   GO; GO:0070062; C:extracellular exosome; IDA:UniProtKB.
DR   GO; GO:0005874; C:microtubule; IDA:UniProtKB.
DR   GO; GO:0015630; C:microtubule cytoskeleton; IDA:HPA.
DR   GO; GO:0043209; C:myelin sheath; IEA:Ensembl.
DR   GO; GO:0003725; F:double-stranded RNA binding; IDA:MGI.
DR   GO; GO:0005525; F:GTP binding; IEA:UniProtKB-KW.
DR   GO; GO:0003924; F:GTPase activity; IEA:InterPro.
DR   GO; GO:0005200; F:structural constituent of cytoskeleton; IEA:Ensembl.
DR   GO; GO:0005198; F:structural molecule activity; TAS:BHF-UCL.
DR   GO; GO:0031625; F:ubiquitin protein ligase binding; IPI:ParkinsonsUK-UCL.
DR   GO; GO:0051301; P:cell division; TAS:BHF-UCL.
DR   GO; GO:0071353; P:cellular response to interleukin-4; IEA:Ensembl.
DR   GO; GO:0030705; P:cytoskeleton-dependent intracellular transport; TAS:BHF-UCL.
DR   GO; GO:0000226; P:microtubule cytoskeleton organization; IEA:Ensembl.
DR   GO; GO:0007017; P:microtubule-based process; TAS:BHF-UCL.
DR   Gene3D; 1.10.287.600; -; 1.
DR   Gene3D; 3.30.1330.20; -; 1.
DR   Gene3D; 3.40.50.1440; -; 1.
DR   InterPro; IPR002452; Alpha_tubulin.
DR   InterPro; IPR008280; Tub_FtsZ_C.
DR   InterPro; IPR000217; Tubulin.
DR   InterPro; IPR018316; Tubulin/FtsZ_2-layer-sand-dom.
DR   InterPro; IPR023123; Tubulin_C.
DR   InterPro; IPR017975; Tubulin_CS.
DR   InterPro; IPR003008; Tubulin_FtsZ_GTPase.
DR   PANTHER; PTHR11588; PTHR11588; 1.
DR   Pfam; PF00091; Tubulin; 1.
DR   Pfam; PF03953; Tubulin_C; 1.
DR   PRINTS; PR01162; ALPHATUBULIN.
DR   PRINTS; PR01161; TUBULIN.
DR   SMART; SM00864; Tubulin; 1.
DR   SMART; SM00865; Tubulin_C; 1.
DR   SUPFAM; SSF52490; SSF52490; 1.
DR   SUPFAM; SSF55307; SSF55307; 1.
DR   PROSITE; PS00227; TUBULIN; 1.
PE   1: Evidence at protein level;
KW   3D-structure; Acetylation; Alternative splicing; Complete proteome;
KW   Cytoplasm; Cytoskeleton; Direct protein sequencing; GTP-binding;
KW   Isopeptide bond; Methylation; Microtubule; Nitration;
KW   Nucleotide-binding; Phosphoprotein; Reference proteome;
KW   Ubl conjugation.
FT   CHAIN         1    451       Tubulin alpha-1B chain.
FT                                /FTId=PRO_0000048108.
FT   CHAIN         1    450       Detyrosinated tubulin alpha-1B chain.
FT                                {ECO:0000305|PubMed:25908662}.
FT                                /FTId=PRO_0000437384.
FT   NP_BIND     142    148       GTP. {ECO:0000255}.
FT   SITE        451    451       Involved in polymerization.
FT   MOD_RES      40     40       N6,N6,N6-trimethyllysine; alternate.
FT                                {ECO:0000269|PubMed:27518565}.
FT   MOD_RES      40     40       N6-acetyllysine.
FT                                {ECO:0000269|PubMed:24906155}.
FT   MOD_RES      48     48       Phosphoserine. {ECO:0000269|Ref.6}.
FT   MOD_RES     232    232       Phosphoserine. {ECO:0000269|Ref.6}.
FT   MOD_RES     282    282       Nitrated tyrosine.
FT                                {ECO:0000250|UniProtKB:P68373}.
FT   MOD_RES     339    339       Omega-N-methylarginine.
FT                                {ECO:0000269|Ref.6}.
FT   MOD_RES     439    439       Phosphoserine.
FT                                {ECO:0000250|UniProtKB:P68373}.
FT   MOD_RES     445    445       5-glutamyl polyglutamate.
FT                                {ECO:0000250|UniProtKB:P68369}.
FT   MOD_RES     451    451       3'-nitrotyrosine.
FT                                {ECO:0000269|PubMed:10339593}.
FT   CROSSLNK    326    326       Glycyl lysine isopeptide (Lys-Gly)
FT                                (interchain with G-Cter in ubiquitin).
FT   CROSSLNK    370    370       Glycyl lysine isopeptide (Lys-Gly)
FT                                (interchain with G-Cter in ubiquitin).
FT   VAR_SEQ     108    223       Missing (in isoform 2).
FT                                {ECO:0000303|PubMed:15489334}.
FT                                /FTId=VSP_055764.
FT   CONFLICT    131    131       G -> R (in Ref. 1; AAA91576).
FT                                {ECO:0000305}.
FT   CONFLICT    290    290       E -> D (in Ref. 1; AAA91576).
FT                                {ECO:0000305}.
FT   CONFLICT    308    308       R -> G (in Ref. 1; AAA91576).
FT                                {ECO:0000305}.
FT   CONFLICT    340    340       S -> T (in Ref. 1; AAA91576).
FT                                {ECO:0000305}.
SQ   SEQUENCE   451 AA;  50152 MW;  94355B4EC2086429 CRC64;
     MRECISIHVG QAGVQIGNAC WELYCLEHGI QPDGQMPSDK TIGGGDDSFN TFFSETGAGK
     HVPRAVFVDL EPTVIDEVRT GTYRQLFHPE QLITGKEDAA NNYARGHYTI GKEIIDLVLD
     RIRKLADQCT GLQGFLVFHS FGGGTGSGFT SLLMERLSVD YGKKSKLEFS IYPAPQVSTA
     VVEPYNSILT THTTLEHSDC AFMVDNEAIY DICRRNLDIE RPTYTNLNRL ISQIVSSITA
     SLRFDGALNV DLTEFQTNLV PYPRIHFPLA TYAPVISAEK AYHEQLSVAE ITNACFEPAN
     QMVKCDPRHG KYMACCLLYR GDVVPKDVNA AIATIKTKRS IQFVDWCPTG FKVGINYQPP
     TVVPGGDLAK VQRAVCMLSN TTAIAEAWAR LDHKFDLMYA KRAFVHWYVG EGMEEGEFSE
     AREDMAALEK DYEEVGVDSV EGEGEEEGEE Y
```

Mascot: http://www.matrixscience.com/
